# Developing an Anxiety Screening Tool for Children in South Africa: Protocol for a Mixed Methods Study

**DOI:** 10.2196/37364

**Published:** 2022-09-27

**Authors:** Fatiema Benjamin, Babatope O Adebiyi, Edna Rich, Nicolette Vanessa Roman

**Affiliations:** 1 Centre for Interdisciplinary Studies of Children, Families and Society University of the Western Cape Bellville South Africa

**Keywords:** South Africa, mental health, anxiety, anxiety screening tool, screening, assessment, early childhood development, child, parent, primary caregiver, teacher, preschool, foundation phase, children, school, youth, development, perception

## Abstract

**Background:**

Early childhood experiences such as trauma, exposure to violence, and poverty can significantly contribute to childhood anxiety, which is viewed as the most common mental health issue among children. In South Africa, there is no uniform tool to screen for anxiety during early childhood. This study aims to develop a tool to screen for anxiety in children aged 4 to 8 years, which could be utilized by preschool and foundation phase teachers to aid in the early identification of childhood anxiety.

**Objective:**

The overall objective of this study is to explore understanding and perceptions of childhood anxiety among teachers, parents, and experts and to develop a tool to screen for anxiety in children aged 4 to 8 years.

**Methods:**

This project will use a mixed method design that will consist of 4 stages. Stage 1 will consist of a scoping review. In Stage 2, data will be collected via semistructured interviews with 60 participants, including parents, teachers, and experts, and will be thematically analyzed. Stage 3 will consist of 20 experts and the researcher collaboratively formulating the proposed screening tool in the form of an e-Delphi component. Once the tool is refined, it will be piloted in Stage 4 with 20 teachers, and data will be analyzed with the Shapiro-Wilk test to test for normality. Additionally, factor analysis will be done to refine and restructure the tool as necessary.

**Results:**

This project was funded from April 2020 to December 2021. Data collection began in September 2022 and is projected to conclude in December 2022 for the qualitative component. The e-Delphi component is expected to be carried out from March to November 2023. Ethical approval was obtained from the Biomedical Research Ethics Committee in November 2021.

**Conclusions:**

Anxiety in early childhood has been linked to various repercussions in adolescence and adulthood, such as school dropout, substance abuse, anxiety disorders, depression, and suicide ideation. Therefore, identifying the presence of anxiety earlier on and providing the necessary referral services could aid in reducing the negative consequences of unidentified and untreated anxiety in early childhood.

**International Registered Report Identifier (IRRID):**

PRR1-10.2196/37364

## Introduction

Early childhood development (ECD) and childhood mental health have received substantial attention since being included in the United Nations Sustainable Development Goals (SDGs) [1]. Across the developmental lifespan, early childhood is the most important developmental phase as it includes and significantly influences the physical, socioemotional, mental, language, and cognitive spheres of development [2]. Therefore, ensuring a healthy early childhood could facilitate the overall development, well-being, and mental health of a child across their lifespan.

In early childhood, mental health is defined as a child’s ability to develop close and secure relationships; to experience, manage, and express a full range of emotions, such as happiness, sadness, frustration, and discomfort; and to explore their environment and learn [3,4]. Mental health problems, which can occur as early as infancy, have been linked to childcare challenges and difficulty in preschool settings, including behavioral problems in school and difficulty learning and engaging with peers and educational content [5]. Anxiety has specifically been identified as the most common mental health challenge in early childhood [3], affecting the child’s social interactions and ability to adequately express emotion. For instance, anxiety can interfere with a child’s ability to transition from a home setting to a school setting. This can result in them displaying aggressive behavior when upset. Although anxiety is a normal response to stressful events [6], when experienced excessively, it results in significant distress and functional impairment [7]. However, there is no universal age-appropriate developmental description of how anxiety presents itself in children, thus contributing to prevalent study findings that suggest few children meet the criteria for anxiety as set out by the Diagnostic and Statistical Manual of Mental Disorders, 5th Edition (DSM-V) [8]. Therefore, it is important to highlight and understand factors contributing to anxiety in ECD to identify how childhood anxiety manifests earlier.

Unrecognized and untreated anxiety symptoms in ECD have the potential to influence the way children interact with their peers, family, and community members [9,10]. Additionally, anxiety symptoms could persist and become more severe, placing children at risk for academic difficulties and school dropout [9,11] as well as the development of anxiety disorders and depression, suicide ideation, unemployment, and substance use disorders later in life [12,13]. However, there is a paucity of literature exploring anxiety during early childhood within the South African context.

To this end, research has found that about half of adults diagnosed with an anxiety disorder had an age of onset before 11 years [9,14], with 50% of cases showing symptoms before 6 years of age [15]. This indicates that anxiety symptoms can be present as early as the ECD phase. Within the South African context, a study conducted by Howard et al [16] found that preschool children (aged 2 to 6 years) showed elevated levels of anxiety symptoms and anxiety proneness compared to children in the Northern Hemisphere. Furthermore, earlier research in the Western Cape, South Africa, found that there was a prevalence between 22% and 25.6% for childhood anxiety symptoms among a sample of 7- to 13-year-olds [17]. This confirms that children in South Africa experience increased levels of anxiety [16]. Therefore, addressing anxiety in ECD is crucial to realizing the 2030 SDGs, specifically Goal 3, which is to ensure healthy lives and promote well-being across all developmental stages.

Significant importance should be placed on identifying anxiety symptoms in young children, which may have a powerful effect on their life trajectories. For example, screening for anxiety early could reduce anxiety-related consequences and improve life-course outcomes by facilitating necessary referrals and treatments. There is a growing body of evidence on educational settings being the most ideal for early intervention. According to Berger et al [18], Yatham et al [19], and Brown et al [20], school is a convenient setting for interventions to take place as it provides the space and opportunity to identify any mental health–related problems, especially for those who are unable to access traditional clinic-based mental health services. Additionally, providing intervention during the early childhood phase has been identified as the most promising and consequential [21]. This may also be related to the substantial amount of time children spend at school and the fundamental role it plays in child development [22]. As such, it would be ideal to screen for anxiety during early childhood within the preschool and foundation phase setting, as it will allow for the early identification of childhood anxiety.

Various international tools have been developed to measure anxiety levels in young children and adolescents. However, the most frequently used tools include the Screen for Anxiety-Related Disorders (SCARED) [23], Spence Children’s Anxiety Scale (SCAS) [24], and the Preschool Anxiety Scale (PAS) [25]. SCARED and SCAS both consist of a child self-report and parent report, whereas the PAS consists of a parent and teacher report. These tools were developed to assess the intensity of anxiety symptoms specifically related to generalized anxiety disorder, separation anxiety disorder, panic disorder, social phobia, and school phobia [22]. SCAS additionally screens for fear of injury and obsessive compulsive disorder [22,26]. The SCARED questionnaire was developed “based on anxiety symptoms in clinical populations” for children 8 to 18 years [27], while SCAS measures anxiety symptoms in children aged 8 to 12 years. Additionally, PAS was developed for children aged 3 to 6 years to assist in clinical assessments and determine whether children are showing higher levels of anxiety [28-30]. Although these tools check anxiety levels in children, there is no tool to screen for the possible presence of anxiety in young children within the unique cultural context of South Africa.

Various cultures and belief systems are present in South Africa that can influence the way mental health is understood and treated. Therefore, understanding cultural influences on mental health can facilitate proper diagnosis and treatment [31]. A study by Booysen et al [32] found that participants understood and described mental illness symptoms in relation to their indigenous cultural perspectives. Thus, culture plays a key role in the conceptualization of how mental health is perceived and understood in South Africa. However, there is no literature that clearly indicates how different cultures in South Africa define anxiety and its symptoms. Therefore, with anxiety being the most common mental health issue in young children, it is imperative to consider the role culture plays in the way children express and experience anxiety as well as the way childhood anxiety is perceived by others. There is limited literature in South Africa that focuses on anxiety in early childhood [16,33,34]. A 2014 report revealed that Western Cape had a 12-month prevalence rate of 17% for mental disorders among children and adolescents [35]. This indicates that there is a significant number of children who have been diagnosed with at least 1 mental disorder within a 12-month period.

Although there are South African studies that have focused on the mental health of children [36,37] and the prevalence of childhood anxiety [38], the literature is scarce, dated, and does not specifically look at anxiety during early childhood. In addition, there is no appropriate tool within the country’s multicultural and multilingual society to screen for the presence of anxiety in early childhood. This makes the availability of a suitable tool that takes cultural factors into account crucial to screen for the presence of anxiety during the ECD phase. Accordingly, this paper describes a process to develop a screening tool for anxiety in children aged 4 to 8 years within the South African context.

## Methods

### Research Design

**Figure 1:**
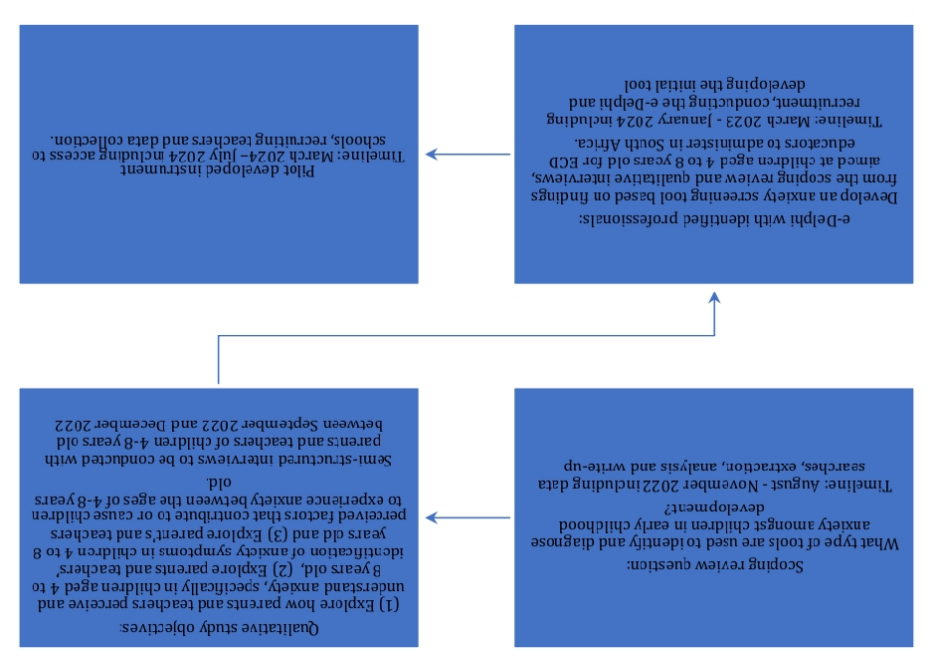
Research process.

This paper aims to outline a protocol for a 4-staged study based on an exploratory sequential mixed method design within a participatory framework. The exploratory sequential design is useful for developing new psychological tests or assessment instruments “based on an initial qualitative analysis and generalizing qualitative findings to a specific population” [39]. In addition, according to Creswell and Creswell [40], combining qualitative and quantitative data provides a deeper understanding of a phenomenon compared to using either approach individually [41]. The participatory action research (PAR) approach, which is a subset of action research, is characterized by participants working with researchers in a collaborative, self-inquiring, and systematic process [42]. It is a cyclical process that is used to explore participant concerns or issues that may impact their lives and aims to bring about meaningful change within communities [43]. The cyclical process includes “exploration, knowledge construction, and action at different moments throughout the research process” [43]. It is therefore applicable to this study, which aims to explore parents’ and teachers’ perceptions and understanding of anxiety and its contributing factors in children aged 4 to 8 years. The principal researcher (author FB) will work in collaboration with participants throughout the tool development process. This will inform and contribute to the development of an appropriate tool to screen for anxiety in children aged 4 to 8 years. Furthermore, it should be noted that parents and stakeholders (eg, teachers and experts) are the respondent groups throughout this research process and that children will not be directly involved. As shown in Figure 1, this research plans to conduct 4 sequential studies, referred to as Stage 1, Stage 2, Stage 3, and Stage 4, wherein the results of one study will guide the subsequent one.

### Stage 1: Identify Available Anxiety Screening Tools for Children Aged 4 to 8 Years

Stage 1 will comprise a scoping review to map the available anxiety screening tools for young children [43] and answer the following question: Which type of tools are used to identify and diagnose anxiety among children in early childhood development? The scoping review will be utilized to inform this study on various formats used for childhood anxiety instruments to identify different components of anxiety in children aged 4 to 8 years and to highlight its use, if any, within the South African context by considering both national and international peer-reviewed literature. This will aid in identifying the importance of a tool developed for circumstances unique to a country or context. Furthermore, this stage will be guided by the PRISMA-ScR (Preferred Reporting Items for Systematic Reviews and Meta-analyses extension for Scoping Reviews) outline. Stage 1 will be used to further inform and strengthen the interview schedules in Stage 2.

#### Inclusion Criteria

Studies that include the use of anxiety screening tools specifically aimed at children will be eligible for inclusion in this review. Further inclusion criteria are studies that (1) include children between the ages of 4 to 8 and their parents, primary caregivers, or stakeholders; (2) are published in or translated to English; (3) peer-reviewed; and (4) use quantitative or mixed methods designs. The time frame will be open as there is little to no available literature in this field. Electronic searches will be conducted using databases such as Academic Search Complete, APA PsycArticles, CINAHL Plus, ERIC, and MEDLINE to search and retrieve literature. These databases were searched in August 2022, and the format of the search strategy differed depending on database requirements (where 1 database required the use of brackets to separate keywords in a search string while another did not). For example, when accessing Academic Search Complete, the following search strategy will be used: (1) childhood anxiety* AND tool OR scale; (2) childhood anxiety* AND instrument OR questionnaire; (3) anxiety in young children OR childhood anxiety* AND teacher scale OR teacher report OR teacher questionnaire; (4) Anxiety in young children OR childhood anxiety* AND parent scale OR parent report OR parent questionnaire; (5) Anxiety in the early years OR anxiety in young children OR anxiety in early childhood development* AND tool OR scale; (6) Anxiety in the early years OR anxiety in young children OR anxiety in early childhood development* AND instrument OR questionnaire; (7) Anxiety in the early years OR anxiety in early childhood development* AND teacher scale OR teacher report OR teacher questionnaire; and (8) Anxiety in the early years OR anxiety in early childhood development* AND parent scale OR parent report OR parent questionnaire.

#### Exclusion Criteria

Studies will be excluded if they are (1) not peer-reviewed, (2) not published or translated to English, (3) include children over the age of 8, and (3) do not use an anxiety scale/questionnaire. Qualitative-only studies and reviews will be excluded because this scoping review aims to map available tools to screen for anxiety in children aged 4 to 8 years.

#### Data Extraction and Analysis

A data extraction tool adapted from Arksey and O’Malley [44] will be used to organize information according to author, year of publication, country of origin, aims and purpose of the study, study population and sample size, methods, and key findings related to the scoping review question.

### Stage 2: Identify and Describe Underlying Behaviors of Childhood Anxiety

This stage aims to establish local relevance and operational definitions of childhood anxiety. This will be done by exploring the different experiences, understanding, and perceptions of childhood anxiety from participants. The outcomes will then be used to assist in the development of the proposed tool in terms of language, concepts, and the cultural applicability.

#### Participants

Participants will be purposively sampled based on the following inclusion criteria to ensure they are appropriate for this study across different stages: (1) parents and caregivers 18 years of age or older (both parents or single parents and caregivers) who have at least 1 child between 4 and 8 years old; (2) teachers (preschool and foundation phase) who actively work with children aged 4 to 8 years; (3) identified experts in childhood anxiety and ECD, including researchers who are well published in the field, psychologists, and social workers; and (3) those who speak English, Afrikaans, or isiXhosa. Parents and stakeholders (teachers and experts) will be included in the qualitative data collection process to obtain a more holistic perspective on childhood anxiety.

#### Data Collection Tool

Semistructured interviews will be used to explore the perceptions of parents and primary caregivers and stakeholders. The interview schedule will consist of 2 parts: (1) demographic information and (2) questions broadly relating to parents’ and stakeholders’ perceptions of childhood anxiety. Interviews with parents will be conducted using interview schedule 1 (Multimedia Appendix 1), and stakeholder interviews will be conducted using interview schedule 2 (Multimedia Appendix 2). These interviews will be guided by the theory, literature, and context and will be conducted in English, Afrikaans, or isiXhosa, as these are the prominent languages spoken in the Western Cape of South Africa [45]. Interviews will be carried out with help from research assistants who are fluent in at least 2 languages, including English. The interview schedule will be piloted with approximately 3 parents and 3 stakeholders to ensure that the questions are easily understood. All interviews are estimated to last between 45 to 60 minutes, and permission will be sought for interviews to be audio recorded.

#### Data Collection

Approximately 60 participants (5 parents and primary caregivers, 3 teachers, and 2 experts from each district in the Western Cape) will be recruited for individual interviews. According to Morse [46], the sample size relatively depends on the quality of the data, with a maximum of 30 to 60 participants being appropriate. However, data collection will cease once saturation is reached. Sampling strategies for parents and primary caregivers will include door-to-door recruitment, snowball sampling, ECD centers, and schools. All COVID-19 safety protocols will be strictly adhered to. These include maintaining a safe social distance during interviews, wearing a mask, and using hand sanitizer before and after the in-person interview. In addition, online platforms such as Facebook, Instagram, and WhatsApp will be used to recruit participants. Similarly, teachers will be accessed through schools, including preschools and ECD centers.

#### Data Analysis

Once data collection is completed and all interviews are transcribed and translated (where necessary), data will be analyzed thematically [47]. Specifically, data will be familiarized by reading and rereading transcriptions, after which initial codes will be generated. Themes will then be searched by sorting the different codes into ideas that are related. Themes will then be identified and named to ensure that meanings are captured and definitions for each theme are generated adequately. Once themes are described and defined, quotations from transcripts will be used to illustrate and capture the essence of the identified themes.

### Stage 3: Develop Initial Instrument

The aim of this stage is to develop a tool to screen for anxiety in children aged 4 to 8 years with the assistance of a professional panel that has knowledge of and experience with anxiety and early childhood. Information gathered from Stages 1 and 2 will also be used to conceptualize childhood anxiety and identify and describe the behaviors that underlie anxiety in children aged 4 to 8 years. In collaboration with the panel, a pool of initial items that reflects the purpose of the screening tool will be generated [48]. Therefore, a Delphi study will be conducted on the panel to facilitate the development of the tool, sequence its items and supporting material, determine the format of the measurement [48,49], and provide feedback on generated items to ensure cultural sensitivity [50]. As such, the cyclical process of PAR will aid in gathering the necessary information to achieve the aim of Stage 3.

#### Participants

Approximately 20 participants will be included in the Delphi study. This includes research psychologists, clinical and school psychologists, or social workers, as well as anyone who has published scientific research related to anxiety and/or early childhood within the South African context. Participants will be purposively selected according to the following criteria: (1) have knowledge of and experience with the topic; (2) are willing to participate; (3) have the time to participate; (4) have effective communication skills [51]; and (5) have least 5 years of experience in the field and are registered with respective professional councils. These criteria will ensure the selection of participants who are experts in the field.

#### Data Collection

An e-Delphi technique will be employed to inform the development of initial items for the tool to screen for anxiety in children aged 4 to 8 years. This online technique will be used for participants to collaboratively engage in the formulation of the proposed screening tool and to review the tool in its entirety. The e-Delphi will consist of online engagements using discussion forums via Google Chat. The participating experts will be asked to discuss the components of anxiety and specify how it may present in early childhood. Following this, initial items will be generated and assessed. Based on the discussions and expert opinions, the principal researcher (author FB) will draft the proposed screening tool and forward it to the panel of experts for review. At this stage, participants will be requested to read each item and comment on its clarity, aesthetics, relevancy, tone, length of time it takes to respond to the item, and cultural sensitivity [50]. The purpose of this is to test for face validity, item validity, sampling validity, outcome validity, and generalizability [50]. This step will be complete once the questions are answered, a consensus is reached, saturation is achieved, or satisfactory information has been exchanged [52]. In the case that participants believe the instrument to be underdeveloped, the principal researcher will go back to the discussion forum and request further assistance from the expert panel on how to improve the screening tool.

#### Data Analysis

Based on participant responses to the open-ended questions accompanying each item on the developed instrument, an inductive thematic analysis will be carried out to analyze the data according to the procedures outlined by Clarke and Braun [47,53].

### Stage 4: Pilot Initial Instrument

In Stage 4, the instrument developed in the previous stage will be tested in a pilot study to determine its validity, reliability, and consistency.

#### Participants

A pilot study will be conducted with preschool and foundation phase teachers to test the screening tool for anxiety in children aged 4 to 8 years. Participants will be purposively selected from ECD centers, preschools, and schools, and teachers in the foundation phase will be specifically targeted. The pilot will be conducted to test the developed quantitative tool in the field and restructure where necessary. Participants will be requested to complete the developed tool to screen for anxiety in children aged 4 to 8 years either in person or online.

#### Sampling

Stratified random sampling will be used in this stage to ensure that the sample is representative of the teacher population, for whom the tool to screen for anxiety in children aged 4 to 8 years is aimed. According to 2016 education statistics in South Africa [54], the Western Cape has approximately 37,518 teachers; therefore, the preliminary sample size of the respondent group is estimated to be 400, rounded off from 395.780, based on the Yamane formula. However, the sample size is subject to change as it is recommended to have an adequate case-to-variable ratio from 5 to 10 participants per item [50]. That said, approximately 67 teachers will be recruited from each of the 6 districts in the Western Cape.

#### Data Collection

Teachers will be requested to use the developed tool to screen for anxiety in children aged 4 to 8 years by considering at least 1 child in their classroom. This will be done to test for item validity, concurrent and predictive validity (criterion-related validity), structure, convergent, discriminant, and divergent validity (construct-related validity), and internal consistency of the scale. In addition, ethical, practical, and cultural issues related to the instrument will be assessed [55] to avoid items inadequately depicting acceptable internal consistency. However, before teachers can complete the developed tool with the consideration of at least 1 child, they will request and receive the consent of both the parent and the child. Once the instrument is completed by participants, it will be entered into SPSS.28 (IBM Corp) software.

#### Data Analysis

Once data collected from the pilot study is captured and cleaned in SPSS.28, test reliability will be conducted to establish internal consistency and to test the assumptions for multivariate statistical analysis. This will be done by conducting a Shapiro-Wilk statistical analysis to test for normality. Item-scale correlations will be used to assess criterion- and construct-related validity and determine how items correlate with each other on the scale. In addition, the Bartlett test of sphericity will be conducted to determine the homogeneity of variance; this tool is recommended to be used before conducting a factor analysis to determine if there is redundancy between identified variables [56]. Additionally, a Kaizer-Meyer-Olkin test will be conducted to determine sample adequacy and the confidence level of the factors in the developed instrument. Based on those findings, a prevalence test will be done, and structural validity will be assessed through exploratory factor analysis to determine the structure of the measure and examine the internal validity of the screening tool. Based on the results, problematic items will be refined or discarded.

### Ethics Approval

Ethical approval (BM21/9/12) was obtained from the Biomedical Research Ethics Committee at the University of the Western Cape. The purpose of the study and its details will be explained to prospective participants, and those who wish to take part will be asked to provide consent before undergoing an interview. Participants will be informed of their right to withdraw from the study at any time without consequence and will be assured that their contribution is completely anonymous. Confidentiality will be ensured by participant names being replaced with pseudonyms or codes. If a participant’s preferred language is isiXhosa, permission will be requested for a research assistant to sit in on the interview for translation purposes. Additionally, audio-recorded files, transcriptions, and raw quantitative data will be password protected and accessible by only the researcher and supervisors. Data will be disposed of by shredding after 5 years. Furthermore, to minimize any potential risk to participants, such as discomfort and psychological or emotional harm, they will be provided with the contact details of relevant referral services, including Families South Africa, Department of Social Development, and Childline. In addition, the principal researcher will gather the telephone number of organizations in areas that offer free counseling services and give them to participants. Furthermore, all COVID-19-related rules and regulations set out by the South African government will be strictly adhered to, such as wearing masks, sanitizing hands, and maintaining social distance. COVID-19 restrictions may affect in-person data collection; therefore, where applicable and feasible, interviews will be conducted via online platforms or telephone. In the case that any participant presents with COVID-19 symptoms, data collection will be ceased to ensure the safety of the participant and researcher. Any disclosure of COVID-19 cases will be treated as confidential. Participants who request further information or assistance related to COVID-19 will be referred to their nearest clinic.

## Results

The project was funded from April 2020 to December 2021. The data collection dates are projected as follows. The scoping review search and screening process began in August 2022, with an expected completion date of November 2022. Qualitative data collection began in September 2022 and is expected to be done by December 2022 and will include recruitment, data collection, and analysis. The qualitative data analysis write-up in preparation for the e-Delphi component is projected to start in March 2023. Furthermore, in collaboration with the e-Delphi participants, who will be recruited in March 2023, the initial tool to screen for anxiety in children aged 4 to 8 years will be formulated between November 2023 and January 2024. The pilot study will take place between March and July 2024 depending on access provided and the availability of schools and teachers. Results are expected to be published in 2024.

## Discussion

### Expected Findings

This study anticipates the identification of available tools to screen for anxiety in young children, specifical children between the ages of 4 and 8. This will be done by conducting a scoping review of peer-reviewed studies that have developed and/or used an anxiety tool for young children. Additionally, the qualitative component of this study will highlight common understanding and perceptions of early childhood anxiety, internal and external factors contributing to anxiety in early childhood, and behaviors associated with anxiety during early childhood. The qualitative component will also consider cultural roles specific to the South African context. The e-Delphi component is anticipated to include experts in early childhood or anxiety to provide professional views and experiences. This will ensure that a holistic perspective is considered in the development of the anxiety screening tool.

Anxiety originating in childhood has been associated with greater severity and comorbidity as opposed to anxiety originating during adulthood [57], and it has been linked to various consequences in adolescence and adulthood, such as school dropout, substance abuse, anxiety disorders, depression, and suicide ideation. Parental psychological control has been identified as a contributing factor, in that parents may transmit anxiety-related fear to their children through verbal communication and overcontrol (eg, telling a child that certain things will scare them or that if they climb too high or run too fast, they will get hurt). Percy et al [57] indicate this by highlighting that the ways in which parents communicate with their young children, both implicitly and explicitly, can increase a child’s anxious behaviors and beliefs. Additionally, other factors have been identified in the etiology of childhood anxiety. These include, but are not limited to, a child’s temperament, maternal psychopathology, family conflict, parenting style, child neglect and abuse, lack of close adult relationships, bullying, rejection, and starting at a new school [58,59]. These factors point to the importance of including childhood mental health in policy and ensuring the accurate execution of interventions.

South Africa is considered to be advanced in developing and implementing health and social policies in comparison to other lower- and middle-income countries. However, a policy analysis by Mokitimi et al [60] revealed that no South African province had a child and adolescent mental health (CAMH) policy or implementation plan to support the National Mental Health Policy Framework and Strategic Plan 2013-2020. This suggests that although a national CAMH policy exists, there is poor implementation across all 9 provinces, pointing to the relevance of this study, which could aid policy making because developing a tool to screen for anxiety in ECD could provide data on CAMH in South Africa. Although there are numerous tools to assess the levels of childhood anxiety and screen for anxiety-related disorders, it is unclear whether a tool already exists to evaluate the possible presence of anxiety in children aged 4 to 8 years, specifically within the South African context.

### Limitations

An identified limitation of this project is that the tool is not aimed to be directly administered to child respondents. However, the proposed screening tool is aimed to be administered by a teacher to determine whether a child shows any signs of anxiety to facilitate early identification and treatment. Another limitation is that this project is not a longitudinal study; therefore, the effectiveness of the tool cannot be monitored over a longer time period.

### Conclusion

Addressing anxiety in ECD is crucial in realizing the 2030 SDGs, specifically Goal 3, which is to ensure healthy lives and promote well-being across all developmental stages. Accordingly, the findings of this study can significantly contribute to the growing body of South African literature on anxiety in ECD, and the developed tool can assist in the early identification of childhood anxiety and provide referrals for treatment. Research findings will be disseminated in the form of a dissertation, scientific publications, and conference presentations. Findings will be presented to participants in the form of a verbal presentation or infographics before the tool is disseminated.

## Appendix

Multimedia Appendix 1 Parents and primary caregiver English interview schedule.

Multimedia Appendix 2 Stakeholders English interview schedule.

## Abbreviations

CAMH: child and adolescent mental health
DSM-V: Diagnostic and Statistical Manual of Mental Disorders, 5th Edition
ECD: early childhood development
PAR: participatory action research
PAS: Preschool Anxiety Scale
PRISMA-ScR: Preferred Reporting Items for Systematic Reviews and Meta-analyses extension for Scoping Reviews
SCARED: Screen for Anxiety-Related Disorders
SCAS: Spence Children’s Anxiety Scale
SDG: Sustainable Development Goal
