# Supplementary material for: Developing an Anxiety Screening Tool for Children in South Africa: Protocol for a Mixed Methods Study
Source: JMIR Res Protoc. 2022 Sep 27;11(9):e37364. doi: 10.2196/37364 (PMC9555325; doi:10.2196/37364)
Supplement: Multimedia Appendix 1 [file resprot_v11i9e37364_app1.docx]

## **Parents/ Primary Caregiver Interview Schedule**

**Demographics**

| **Gender/** *Geslag*/ Isini | Male/ *Manlik/* Indoda | | |  | | | | | Female/ *vroulik/* Ibhinqa | | | | |  | |
| --- | --- | --- | --- | --- | --- | --- | --- | --- | --- | --- | --- | --- | --- | --- | --- |
| **Your Age/** *jou ouderdom/* Iminyaka yakho |  | | | | | | | | | | | | | | |
| **Your CHILD’S Age /** *Jou Kind se ouderdom/* Iminyaka yomntwana wakho |  | | | | | | | | | | | | | | |
| **Highest education level completed/** *hoogste onderwysvlak voltooi* **/**Inqanaba lemfundo onalo | Primary school / *laerskool/* Isikolo samabanga aphantsi | | Some high school | | | Completed high school | | | Some college/University | | | Completed University | | | Postgraduate studies |
|  |  | |  | | |  | | |  | | |  | | |  |
| **Post matric education or training/** *na matriek onderrig of opleiding/* Inqanaba lemfundo ephakamileyo onalo | None /  *Geen/* Alikho | Certificate/  *Certifikaat* | | | | | Diploma/  *Diploma* | | | | | | Degree/  *Graad* | | |
|  |  |  | | | | |  | | | | | |  | | |
| **Race/** *ras/* Uhlanga | Coloured/ Kleurling | | | | Black/  *Swart* | | | White/  *wit* | | | Indian / Asian | | | | |
| **Home language/**  *Husitaal/*  Ulwimi lwasekhaya | Afrikaans | | | | English | | | isiXhosa | | | Other | | | | |
| **Who is the head of your home? /** *wie is die hoof in die huis?/*  Ngubani intloko yomzi? | - Me/ *Ek/* Ndim - My spouse or partner/ *My eggenoot of lewensmaat/* Ngumlingani wam - My mother/ *My ma/* Ngu Mama wam - My father/*My pa/*  Ngu Tata wam - My grandmother/ grandfather/ *my ouma/ oupa/* Ngu Makhulu okanye Takhulu wam - Member of extended family (aunt/uncle/cousin)/ *lid van die uitgebreide familie (tante/ oom/neef/niggie/* Sisihlobo sam (malume/makazi/mzala) | | | | | | | | | | | | | | |
| **What is the structure of your family?/**  *Wat is die struktuur van jou gesin/*  Lume njani usapho lakwakho? | Married/ *getroud/*  Sitshatile | | Living together but not married/ *leef saam maar nie getroud nie/*  Sihlala sonke kodwa asitshatanga | | | Single, do not live together and are not married/ *enkleloopend, maar bly nie saam nie en nie getroud nie,* asitshatanga kwaye asihlali sonke | | | Single because he / she is widowed/ *enkleloppened want hy/sy is ‘n weduwee/*  Andinamlingani kuba ndingumhlolokazi/mhlolo | | | Single because he / she is divorced/ *enkleloopend want hy/sy is geskei*/ Andinamlingani ngoba ngoba sawuqhawula umtshato | | | Extended family/ *uitgebreide gesin/familie/*  Ndihlala nezihlobo zam zangaphandle |
|  |  | |  | | |  | | |  | | |  | | |  |
| **Area that you live** / *Gebied waarin u woon*: / Indawo ohlala kuyo: | Urban Area/Dolophini | | | | | | | Rural Area/Ezilalini | | | | | | | |
| **What is the name of the area that you live in?** / *Wat is die naam van die omgewing waarin u woon?* / Ngubani igama lendawo ohlala kuwo? |  | | | | | | | | | | | | | | |
| **How many children in the home? /** *hoeveel kinders in the huis? /* Bangaphi abantwana ekhaya/endlini? |  | | | | | | | | | | | | | | |
| **Are you currently working? /** *werk jy tans? /* Ingaba uyasebenza**?** | - Yes/ *Ja/* Ewe | | | | | | | | - No/ *nee/* Hayi | | | | | | |
| **What is your occupation? /** *wat is jy beroep/*  Yintoni msenzi wakho? |  | | | | | | | | |  | | | | | |

1. To your understanding, what is anxiety or feeling anxious?
   1. How would you describe anxiety? (Specifically, anxiety in children). Probe: what does it look like when someone feels anxious or has anxiety? What are the words that you would use to describe someone feeling anxious or experiencing anxiety?
2. Do you think young children can have/ experience nervousness? Please elaborate on why you think so or don’t think so?
3. To your understanding, what does anxiety present/ look like in young children between ages 4-8 years?
4. Overall, what do you think contributes to anxiety in children? Why? (What causes child anxiety? At school, at home or community). In general.
5. Do you think your child is a ‘nervous’/ anxious child? What do you think makes them feel this way? (Probe: How do you react to this?)
6. How often does your child worry? Do you often see your child fidgety, fearful, distressed, scared, uneasy? What makes them feel this way? Could you please describe/explain? How often does this happen? Per week, per month? To your knowledge, how long has this been happening?
7. Is it difficult for your child when he/she is away from you? (How does your child react when you must leave him/her?) Why?
8. Describe the behaviour of your child when he/she experiences different emotions? (Specifically ask how they behave when they are happy, when they are sad, and when they are angry? Or talking to people they are unfamiliar with?)
9. Do you think that your children experiences stress or stressful situations? Why?
10. How would you say your child interacts with others? (Their friends, family members, teachers, when meeting new people for the first time?)
11. How would you describe your child’s behaviour when he/she started school? Describe any differences in his/her behaviour since then. Tell me about the times when your child/ren had to meet their new teacher/ class friends at the beginning of the year?
12. How would you describe your child’s behaviour outside of school? Have you noticed a difference or has the teacher described behaviour of your child that you were not aware of?
13. To your knowledge, how does your child feel about school/group work/speaking in front of others? (How does your child behave if they have a test, exam, or oral presentation?).
14. Do you have any helpful tips for other parents who may think that their child has anxiety? (What signs should they look out for and what should they do?).
15. Do you think getting professional mental health assistance will benefit parents and their young children? Could you please explain?
16. What kind of assistance do you think is most helpful?
