# Supplementary material for: Developing an Anxiety Screening Tool for Children in South Africa: Protocol for a Mixed Methods Study
Source: JMIR Res Protoc. 2022 Sep 27;11(9):e37364. doi: 10.2196/37364 (PMC9555325; doi:10.2196/37364)
Supplement: Multimedia Appendix 2 [file resprot_v11i9e37364_app2.docx]

## **Stakeholder Interview Schedule**

**Demographics**

| Date: |  | Time: |  |  |
| --- | --- | --- | --- | --- |
| Gender:/ Geslag: | Male | Female | Other |  |
| Age:/Ouderdom: |  |  |  |  |
| Race:/Ras: | Coloured | Black | White | Indian/Other |
| Home language:/Huistaal: | English | Afrikaans | isiXhosa | Other (specify) |
| Highest grade/standard passed:/Hoogste graad/standard geslaag: | Primary school | High school | Certificate/higher training | Diploma/Degree |
| Employment:/Indiensneming: |  |  |  |  |
| Duration of employment:/Duur van diens: |  |  |  |  |

1. Please could you name and describe your position at your workplace?
2. What is your understanding of anxiety? (What is your understanding of childhood anxiety?)
   1. Do you think they can have/experience anxiety? Please elaborate.
   2. At what age do you think children generally start showing anxiety symptoms?
   3. How would you say anxiety manifests in children 4-8 years old?
3. To your understanding, what factors contributes to children experiencing anxiety? And what is it commonly associated with?
   1. What do you think makes children ‘nervous’/anxious?

**(If the social context is not included in response to question 3, ask question 4)**

1. Can the living situation of a child contribute to them having anxiety? Why/How? (What factors in the community, school, and home can lead to child anxiety?).
2. Do you think parents are able to identify anxiety-related challenges in their children? Please explain.
   1. What would be needed to further improve parents' and teachers’ perceptions, attitudes and understanding of child anxiety?
3. Do cultural beliefs and values influence how anxiety is viewed and treated? Please explain. (How does this apply to anxiety in children?)
4. Can you please tell me about the most common behaviours of anxious children?
   1. For example, behaviours in the classroom, talking to people they are unfamiliar with, among peers, in gatherings, being away from their parents etc.
5. Is there any assistance available to determine whether a child displays a presence of anxiety symptoms? For example, if an adult were to be suffering from anxiety, they would seek therapy. To your knowledge, what is available for children displaying anxiety symptoms?
6. What do you think is needed for teachers, to assist them to identify anxiety-related problems in children?

**ASK TEACHERS ONLY:**

1. What are some of the common challenges or problems that teachers face when there are anxious children in the classroom? For example, a child who does not interact with peers or actively participate in classroom activities.
2. To your knowledge, how does a child (or children) that you suspect suffers from anxiety behave when they must do group work or speak in front of others within the classroom?
   1. How does the child behave when they have a test or have to do an oral either to you or in front of their peers?
